# Is Digital Healthcare Better Than None? The Digital Health Technology Dilemma in Low-Income Countries

**DOI:** 10.1007/s41649-025-00395-9

**Published:** 2026-05-05

**Authors:** Brandon Ferlito, Heidi Mertes

**Affiliations:** Bioethics Institute Ghent, Department of Philosophy and Moral Sciences, Faculty of Arts and Philosophy, Ghent University, Ghent, Belgium

**Keywords:** Digital health technologies, Low-income countries, Early access programmes, Ethics and regulation, Health equity, Accountability

## Abstract

Digital health technologies (DHTs) are often promoted as solutions to healthcare access challenges in low-income countries (LICs), where a lack of infrastructure, healthcare professionals, and regulatory capacity makes them especially appealing. In such contexts, DHTs can provide services that would otherwise remain out of reach. Yet their implementation raises significant ethical and regulatory concerns. These concerns are heightened in LICs, where technologies designed in high-income countries may perform less reliably, accountability is weak, and corporate interests often dominate development and governance. This paper compares DHTs with Early Access Programmes (EAPs) for investigational therapies, both of which involve offering imperfect interventions under conditions of uncertainty. While EAPs are tightly regulated, DHTs are often introduced with limited oversight and marketed as final solutions. Unlike pharmaceuticals, which carry immediate risks such as severe side effects, the risks associated with DHTs are typically indirect or delayed, manifesting through misdiagnoses, breaches of privacy and confidentiality, digital inequalities, and the erosion of trust in healthcare systems. Using examples from Uganda and Malawi, the analysis highlights how permissive adoption risks promoting a ‘better than nothing’ standard of care, while rigid regulation may deny immediate benefits. We argue for a tiered approach: conditional, context-sensitive oversight that expands access without normalising lower standards of care, embedding privacy, confidentiality, equity, and accountability as central design criteria.

## Presenting the Dilemma

Imagine an individual in a low-income country (LIC)^1^ living with hypertension, facing limited access to healthcare facilities, high transport and medication costs, and social barriers to seeking care. A digital health technology (DHT), such as a solar-powered blood pressure monitor linked to a simple mobile app, can send daily readings to a cloud platform and prompt timely intervention from a healthcare professional (HCP). This illustrates the promise of DHTs: bridging gaps in healthcare access where traditional systems are limited or absent (Pioch et al. 2024).

Of note, we focus on LICs because these settings face particularly acute shortages in healthcare infrastructure, limited regulatory capacity, and high donor dependency—factors that make DHTs especially appealing in these contexts (Badr et al. 2024). At the same time, it is important to acknowledge that LICs are far from homogenous. While they are grouped together for analytic convenience, they differ significantly in terms of infrastructure, health system capacity, political priorities, and the degree of donor dependency. To bring our arguments across without over-generalisation, we use short comparative examples (i.e. Uganda and Malawi) throughout the paper as illustrations rather than exhaustive cases.

Nonetheless, DHTs^2^ primary appeal in LICs lies in their apparent ability to overcome geographic, systemic, and economic barriers to healthcare (McCool et al. 2022; World Health Organization 2019) . For many, they are not just convenient add-ons but sometimes the only way of accessing healthcare (Gayesa et al. 2023; Geldsetzer et al. 2022). At the same time, DHTs raise unresolved ethical concerns. These include questions of safety and effectiveness (Grosman-Rimon and Wegier 2024), algorithmic bias (Mellino et al. 2022), privacy and confidentiality, the deepening of the digital divide (Badr et al. 2024), and the risks of transferring large volumes of health data to private tech firms driven by commercial interests rather than public good (Salmon and Thompson 2021). There are also blurred boundaries between medical devices and lifestyle products (Lievevrouw et al. 2021), liability when harm occurs (Bottomley and Thaldar 2023), and gaps between the empowerment rhetoric and the actual digital literacy required to enable that empowerment (Gray et al. 2024). These tensions explain why DHTs have been cautiously integrated into formal healthcare systems but have spread rapidly in lifestyle and wellness domains (Yan et al. 2021). Furthermore, these challenges, particularly the risks to privacy and confidentiality, the deepening of the digital divide, and issues of accountability and responsibility are not peripheral but central to our analysis. We return to them later in the paper, since they pose some of the most urgent ethical questions for DHT use in LICs.

Concerns about the risks of DHTs are heightened in LICs, as these technologies often perform less reliably when used outside the conditions or datasets in which they were developed (Sendra-Balcells et al. 2023; Yang et al. 2023). This means that tools designed and tested in high-income countries (HIC) may produce weaker results when transferred to very different clinical, cultural, or infrastructural contexts. Structural power imbalances add another layer of vulnerability. Companies that design and control DHTs typically operate from HICs, while users in LICs have little bargaining power or regulatory protection (Siala and Wang 2022). Scholars have described this as a form of algorithmic colonisation: the imposition of HICs’ values and assumptions through technologies deployed in LICs (Birhane 2020). It may, however, seem reasonable to argue that, since LICs have so few alternatives, DHTs should be allowed even if they do not meet the same standards required in HICs and even if they present heightened risks in LICs.

There seem to be two possible reactions to this predicament. The first is to argue that we are asking the wrong question altogether: what looks like a technological problem is really a matter of justice. Health disparities between HICs and LICs are not incidental; they are sustained by global economic structures that prioritise profit over equitable access—a neoliberal approach to healthcare (Baru and Mohan 2018). The second response is to acknowledge this injustice, but to defend the introduction of DHTs as a partial amelioration of the situation: it may be justified to apply different standards for DHTs in different contexts, provided they still deliver concrete improvements. This ‘context-sensitive’ view rests on the principle that *ought implies can*: the claim that only the best standard of care is compelling only holds when that level of care is realistically achievable. As Benatar argued in relation to clinical trials in developing countries:

[…] it must surely be agreed that standards of care during clinical trials should neither be set at levels that are impossible to achieve, nor should research subjects be denied higher levels of care than may be available within their country if much higher standards can be achieved during the research process (Benatar 2002, 1136).

Benatar acknowledges that unequal standards are unfair and that structural injustices must ultimately be remedied. Yet, in the absence of macro-level change, he urges micro-level action to reduce disparities as much as possible. Applied to DHTs, this means their desirability should be judged by whether they offer genuine improvements *on the ground* for people in context, rather than by comparison with ideal but unavailable alternatives.

Both reactions are problematic. The justice-first stance risks sacrificing tangible improvements in healthcare access for victims of structural and systemic injustice, while the context-sensitive stance risks settling for second-best solutions and accepting the status quo as permanent. In order to move beyond this dichotomy, this paper will draw on a useful comparison with Early Access Programmes (EAPs) for investigational therapies. In EAPs, patients with no remaining treatment options may access experimental drugs before full regulatory approval, but only under strict conditions: informed consent, available safety data, and supervision by HCPs (Raus 2016). Both DHTs in LICs and EAPs involve offering imperfect solutions under uncertainty, but EAPs operate within structured regulatory frameworks, while DHTs often lack such oversight and are marketed as final solutions. For example, in Uganda, telemedicine platforms scaled rapidly during COVID-19 to fill urgent care gaps, yet reports highlighted unclear service costs and weak data privacy protections (Sekalala et al. 2025).

What starts as ‘emergency’ measures often becomes permanent, kept in place by powerful companies and political convenience. This also indicates a bigger risk of techno-solutionism and ‘pilotitis’ (Huang et al. 2017), where quick fixes end up replacing—rather than supporting—real investment in public health systems. While DHTs and investigational therapies are clearly distinct, they share a key ethical question: should individuals with no or few other valid alternatives have access to interventions that are not yet fully vetted? In both cases, patients face a lack of established, effective options and must decide whether to accept potential risks in exchange for possible benefits under conditions of great uncertainty. These risks are discussed in detail in subsequent sections.

The central contribution of this paper is to argue that the governance of DHTs in LICs must avoid the false choice between permissive adoption and rigid regulation. By comparing DHTs with EAPs, we show how conditional and structured oversight can expand access without locking LICs into second-best healthcare standards. This comparison also highlights how heterogeneity across LICs shapes both the risks and responsibilities at stake. In doing so, we move the debate beyond the notion of ‘better than nothing’, toward a framework that integrates short-term gains with long-term justice-oriented commitments.

We start first by outlining the opportunities and risks of DHTs in these settings, then use EAPs as a point of comparison. We offer this comparison as a heuristic device to stimulate discussion on how differing regulatory landscapes shape healthcare interventions, rather than as a perfect model to be followed. Three themes shape our discussion: (1) relative advantage and structural injustice, (2) trust, risk, and blurred lines, and (3) regulation and liability. Throughout the paper, we turn to wider concerns such as the digital divide (digital inequalities), privacy and confidentiality, issues of accountability and responsibility, and the risk of reinforcing existing inequalities. The paper then concludes by suggesting a practical oversight model for DHTs in LICs—one that allows short-term access but avoids accepting a ‘better than nothing’ standard of healthcare.

## The Role of Digital Health Technologies in LICs: Opportunities and Risks

DHTs can expand access in LICs, but their rapid and often unregulated adoption raises serious ethical risks. Unlike pharmaceuticals, which face rigorous checks, many DHTs enter markets with minimal oversight (Woulfe et al. 2022). This regulatory gap can expose patients to misdiagnosis, unsafe treatment, or algorithmic bias (Weissglass 2022), just to mention a few, while debates continue over whether stricter rules would protect or hinder access (Postigo 2023).

Data privacy, confidentiality, and security are also major concerns, as DHTs generate sensitive health data, often stored on privately owned cloud servers (Spigel et al. 2018; Zandesh 2024). In many LICs, absent, weak, or inconsistently enforced data protection laws leave patients exposed to breaches of confidentiality (McCool et al. 2022). In Uganda, for example, a report by the *Collaboration on International ICT Policy for East and Southern Africa (CIPESA)* notes that although a Data Protection and Privacy Act was passed in 2019, a personal data protection office was only operationalised in 2021, and oversight of digital health innovations has remained limited. The report highlights capacity gaps at the facility level, inadequate standard operating procedures (SOPs), and weak enforcement of data protection standards, factors that pose serious risks to patient privacy and confidentiality (CIPESA 2023).

In terms of distributive justice, while DHTs have the potential to increase access to healthcare, it inherently benefits those who already possess smartphones, stable internet, and basic technological literacy (Biga et al. 2024). According to Heeks (2022), such ‘access’ can exist alongside harmful digital inclusion, where powerful actors gain disproportionate benefits from the data, work, or resources of less-advantaged groups. The reliance on user-owned connectivity and data packages also often shifts costs onto the most vulnerable, making access contingent not only on device ownership, but also on the ongoing affordability of digital participation. This means pushing costs and risks onto users, usually in ways that are hidden and shaped by unequal power. In this sense, what is often framed as a ‘digital divide’ is better understood as ‘digital inequalities’—systematic patterns of exclusion that layer on top of existing socio-economic hierarchies and, crucially, patterns reproduced through inclusion via exploitation, surveillance, taking shared resources away from communities, and controlling access through data (Heeks 2022; Lutz 2019).

As Withers (2021) argues, these inequalities are not merely about who gains access, but about how the very outcomes of DHT implementation are distributed. Again, Uganda provides a concrete example: a study by Mwandacha et al. (2024) found that women with mobile phone access were significantly more likely to report attending healthcare outreach visits (87.3% vs. 72.6%) and visiting health centres (96.9% vs. 93.5%) compared to those without access. While these findings highlight the potential of mobile access to facilitate engagement with healthcare, they also expose how DHTs risk amplifying inequality divides. At the same time, because this was a cross-sectional study, it cannot show that phone access itself causes higher healthcare use. Wealthier or better-educated women may both be more likely to own phones and more likely to seek care, which means the gap could partly reflect existing socio-economic inequalities rather than a direct effect of digital access.

It is important to recognise that these inequalities predate DHTs: they reflect broader structural and systemic inequalities in global health (Baciu et al. 2017), driven by underfunded public systems, the medical brain drain, and pharmaceutical monopolies. In this light, DHTs can look less like solutions and more like market-driven stopgaps that commodify scarcity while leaving the root causes intact (Baru and Mohan 2018; Birn et al. 2017; Schüklenk and Ashcroft 2002).

Additionally, the rapid expansion of DHTs has been driven largely by private-sector innovation rather than public health initiatives (Goodman et al. 2024; McCool et al. 2022), and governments often provide support to facilitate their growth. The European Commission (2025), for example, states that a goal of the European Health Data Space Regulation is to ‘[foster] a health-specific data environment that supports a single market for digital health services and products’. While the private sector can supplement state-led healthcare in LICs, there is great concern over their potential negative impacts on public health governance as states shift healthcare responsibilities to the market (Dove et al. 2025; Suarez-Villa 2012). As digital health markets consolidate into the hands of a few powerful corporations, it is clear that DHTs are not politically neutral: they raise direct questions about who designs healthcare systems, who profits, and who carries the costs when things go wrong (Gotsadze et al. 2024; Suarez-Villa 2012). Governments should therefore not accept private-sector innovations as is; they should actively steer them in line with national healthcare strategies.

The private investment model is, however, unlikely to achieve health equity, which, as Arnaout et al. (2023, 2,3) point out, ‘… is a complex multi-sectoral issue which requires multiple stakeholders to address’ and ‘… requires partnership with those who are attuned to the particular needs of the local population and have a long-standing trusted relationship with the community’. The increasing reliance on corporate-led DHTs suggests that health is being reshaped as a product of innovation cycles, rather than recognised as a basic human right. Yet, even in state-led healthcare systems, economic and political factors have long shaped healthcare delivery, from drug pricing to hospital funding (Jansky et al. 2024). The key issue is not whether healthcare can be fully separated from markets, but whether DHTs support or undermine efforts to strengthen public health. The challenge, then, is to use DHTs without letting their usage justify neglect of traditional healthcare systems or excessive reliance on the private sector.

Some policymakers frame DHTs as interim measures while LICs build infrastructure, but as the COVID-19 experience showed, ‘temporary’ solutions often become permanent due to corporate power and political expediency. Uganda’s experience during COVID-19, where multiple digital health tools were rapidly deployed and many normalised into routine use, illustrates how ‘temporary’ fixes can quickly become institutionalised (Kabwama et al. 2024) While these tools provided valuable functions during the crisis, their roll-out raises questions about governance, donor influence, and the quiet substitution of structural and systemic investment with technological stopgaps. A similar dynamic can be seen in Malawi’s Chipatala Cha Pa Foni (Health Centre by Phone) hotline. Initially launched as a donor-driven pilot and scaled up during the pandemic, the service was eventually adopted nationally as a core component of the health system (van Niekerk et al. 2023).

Problematizing such examples reveals the risks at stake: DHTs may shift the standard of care, normalising ‘good enough’ services for marginalised populations while diverting political will and resources away from broader infrastructural reform. What appears as innovation, then, may also reinforce hierarchies of access, embedding unequal health futures under the guise of progress. This means safeguards are needed to ensure DHTs complement, rather than replace, structural and systemic investment. Iris Marion Young’s (2011) concept of collective responsibility provides a helpful way to frame this. Structural injustices persist because individuals and institutions act within established norms and systems. Everyone connected to these injustices carries a duty to challenge and transform them. From this perspective, the key question is not simply whether DHTs should be embraced or resisted, but how their introduction might become a lever for broader reforms in global health governance: how can DHTs be rolled out in LICs in ways that reduce structural and systemic harms rather than reinforce them?

To explore this, we turn to the case EAPs where similar dilemmas about imperfect interventions are offered under conditions of uncertainty. Looking at EAPs allows us to ask whether DHTs in LICs represent pragmatic stopgaps, dangerous precedents, or opportunities to reimagine global health structures.

## Comparing Digital Health Technologies and Early Access Programmes

DHTs and EAPs share ethically relevant features: both offer uncertain benefits, partly predictable risks, and are often provided by private actors to vulnerable groups with limited alternatives. Yet important differences remain: EAPs address life-or-death situations, while DHTs usually do not, and the vulnerabilities at stake in LICs are largely products of structural inequities. The morally relevant similarities—uncertain benefits, partly known risks, private market interests, and patient vulnerability—make the parallel worth exploring, even as important dissimilarities remain. Using three dimensions—(1) relative advantage and structural injustice, (2) epistemic trust, risk, and blurred lines, and (3) regulation and liability—this section asks whether the safeguards in EAPs can inform a more responsible framework for DHTs in LICs.

### Relative Advantage and Structural Injustice

Both DHTs and EAPs aim to, at least temporarily, address unmet healthcare needs when no alternatives exist, operating under the principle of relative advantage: the idea that an imperfect solution may still be preferable to none (Rogers 1981). However, while they may share this ethical justification, they diverge significantly in their long-term impact and potential risks. A core ethical concern is whether the advantages of DHTs justify their risks, especially in a context where weak regulatory oversight is combined with a lack of awareness of the limitations and risks of the technology by its users (Mittelstadt 2017; Nasir et al. 2023). While EAPs are designed as temporary solutions in anticipation of extensively vetted treatments (Putter et al. 2019; Raus 2016), DHTs are often marketed as if they were final solutions with few risks, yet they frequently enter the market with minimal oversight and limited clinical validation (Mathews et al. 2019).

The increasing focus on DHTs in LICs also carries the risk of being used to justify underinvestment in traditional healthcare infrastructure. Governments and global health organisations may treat DHTs as low-cost substitutes, rather than as complements to in-person services. If these innovations follow the curve of Christensen’s et al. (2000) concept of disruptive innovation in healthcare, which suggests that low-cost alternatives which are initially of poorer quality than established care can disrupt and increasingly replace the traditional healthcare market while their quality improves, this would not be problematic. However, it remains uncertain whether DHTs will truly follow this paradigm and the label of Christensen’s concept can only be assigned in hindsight. There are many new low-cost ‘innovations’ that never succeed in overthrowing a market and becoming a more accessible replacement for existing solutions, while meeting the same quality standards as traditional care. It is to be expected that many DHTs are not here to stay, let alone radically transform healthcare. Again, Uganda provides a telling illustration: by the early 2010s, multiple donor-backed digital health projects flooded the scene, prompting a formal moratorium by 2012 to demand better coordination, interoperability, and sustainability (Huang et al. 2017). This illustrates how *pilotitis* arises—when donor-driven digital health projects multiply rapidly but remain small, fragmented, and unsustainable. Rather than strengthening systems, they create temporary individual fixes that expand access momentarily but fail to produce long-lasting structural improvements (Bhatia et al. 2020).

Similar dynamics are evident in Malawi. A comprehensive review of mHealth projects (2010–2017) found that most DHTs’ pilots never scaled. Reported reasons for this were not that they were replaced by more structural solutions, but rather a lack of government backing or long-term planning. Experts have since proposed implementation models to address critical deficits in infrastructure, governance, and coordination (Malanga and Chigona 2018). While well intended, the downside of such investments, however, is that they solidify temporary solutions and divert resources and attention from other healthcare interventions. This aligns with a critique of disruptive innovation in sociological circles. Critics such as Lepore (2014) and King and Baatartogtokh (2015) argue that the idea of disruption often acts as a normative ideology over time, potentially justifying trade-offs that perpetuate inequalities and lower healthcare standards, diverting investment from traditional healthcare without delivering comparable outcomes. This further raises concerns about whether DHTs are truly improving healthcare access or entrenching a two-tiered health system, in which LICs are effectively left with lower standards of healthcare than HICs (Badr et al. 2024; Hurt et al. 2015). While relative advantage provides a convincing justification for early adoption, DHTs currently operate outside the strict frameworks that apply to EAPs, making their risks more difficult to mitigate.

### Epistemic Trust, Risk, and Blurred Lines

Both DHTs and EAPs blur the line between experimentation and healthcare provision, operating in uncertain healthcare spaces where patients and users of such interventions and technologies must trust the knowledge, intentions, and authority of those developing and implementing these solutions, without having any guarantees of an overall benefit or adequate safety measures. The lack of evidence-based practices raises questions about possible risks, unintended harm, and the long-term safety of patients. In both cases, individuals rely on solutions that have not undergone full validation. However, trust in these interventions is shaped by different factors. In EAPs, uncertainties are explicitly acknowledged, and patients make informed decisions under a HCP’s guidance (Fountzilas et al. 2018). In contrast, DHTs are frequently adopted without clear communication about risks or with only minimal oversight. This distinction means that while EAP patients place epistemic trust in a single, high-risk decision, users of DHTs are asked to place ongoing trust in technologies that lack the same transparency about limitations or safeguards against risks.

During the COVID-19 pandemic in Uganda, telemedicine platforms expanded access to care in rural areas, yet patients were rarely informed about service limitations or data protections (Sekalala et al. 2025). Trust was fostered through urgency and necessity, but without transparency, leaving users to bear risks they could not fully anticipate. Beyond trust in clinical accuracy, trust also hinges on the protection of sensitive health information. In many LICs, however, data protection laws are often underdeveloped or unevenly enforced, exposing patients to risks when digital systems fail or when their data are shared without their informed consent (Bouke et al. 2023; Mengiste et al. 2023). For individuals with stigmatised conditions such as HIV, breaches of confidentiality can produce not only loss of trust but also serious social harms, including discrimination, exclusion, or even violence. In such contexts, safeguarding privacy and ensuring confidentiality is not simply a matter of regulatory compliance; it is a matter of justice and patient safety.

As Orii et al. (2024) found, privacy concerns deeply affect trust and digital health uptake among HIV care clients in Malawi. Participants reported fears that their HIV status might be disclosed without consent, particularly in small communities where breaches could quickly lead to stigma and discrimination. They expressed uncertainty about who exactly had access to their records—distinguishing trusted HCPs from non-medical staff—and some worried about how data shared with government agencies or donors might be used. These concerns shaped their trust in the health system and their willingness to engage with DHTs. In this way, weak privacy and confidentiality safeguards not only expose users to harm but also directly undermine the willingness to adopt potentially beneficial technologies, illustrating how data governance and trust are inseparable from equitable digital health implementation.

Furthermore, unlike EAPs, which are delivered by HCPs within clinical settings, DHTs in LICs are often adopted outside formal healthcare structures, being directly available on a mobile device, for example. Their uptake may be shaped by local community networks, recommendations from peers or family, community health initiatives, or prior positive experiences (Kansiime et al. 2024; Vaghefi and Tulu 2019; Yi et al. 2024). Another challenge with DHTs is that trust can be shaped in ways that may not serve users’ interests—for instance, through manipulation or commercialisation, particularly when corporate interests drive digital health implementation in LICs (Kickbusch et al. 2021). DHTs, in comparison to EAPs, are developed by private tech companies that may prioritise user engagement over clinical effectiveness. This creates the potential for profit-driven incentives to influence how trust is cultivated, with some platforms relying on paid add-ons or data-driven business models that put patients at risk of misplaced trust (Bak et al. 2025; Graham 2023).

Malawi tries to legislate against these dynamics: its Digital Health Strategy (2020–2025) emphasises privacy, security, and adherence to SOPs for data access, release, and disaster recovery, while even tracking the proportion of digital health systems that host their data locally. However, the strategy itself acknowledges significant implementation gaps, noting problems such as the lack of sustainability and limited coverage of digital health solutions, weak interoperability between systems, low trust in data quality and use, and poor coordination across digital health efforts (Malawi Ministry of Health 2020). This reveals a central tension: the classic ‘placebo policies’ problem (see Head 2022). While the policy projects an image of robust governance, the conditions required to enact such protections remain fragile. In this sense, the privacy, confidentiality, and security provisions risk functioning more as symbolic assurances rather than as enforceable safeguards. The strategy highlights ‘best practice’ goals without the capacity to achieve them, which risks giving digital health expansion a false sense of legitimacy and safety, while leaving patients exposed to the very risks it aims—and claims—to prevent.

### Regulation and Liability

EAPs often function within a structured legal and procedural framework, ensuring that investigational therapies meet minimum safety and efficacy thresholds before being made available to patients. Interventions entering EAPs must typically have passed Phase I trials, which establish safety, and Phase IIa trials, which provide preliminary evidence of efficacy (Raus 2016). These regulatory steps ensure that while the investigational therapy remains experimental, it has undergone some degree of validation before reaching patients. In contrast, many DHTs in LICs enter the market without equivalent regulatory checks, often lacking clinical testing, standard approval pathways, or independent oversight (Bene et al. 2024). Unlike pharmaceuticals, which pose immediate and direct risks, such as severe side effects or life-threatening complications, the risks associated with DHTs tend to be indirect or delayed, but they can still be significant, especially when DHTs are rolled out on a wide scale.

This risk can manifest over time through misdiagnoses, privacy and confidentiality breaches, increasing digital inequalities, loss of trust in healthcare systems, economic burdens, and behavioural or psychological effects. In Malawi, the introduction of biometrics and electronic health records has raised patient concerns about data privacy and confidentiality, with anxieties about who might access sensitive information shaping their willingness to engage with these tools. At the same time, the wider digital health landscape in Malawi and across the region remains fragmented and poorly integrated, with weak interoperability and governance undermining data quality and coordination (Ibeneme et al. 2022; Mwapasa et al. 2020). These factors may not trigger immediate crises, but over time, they steadily erode trust and compromise the systems meant to strengthen care.

Moreover, one of the largest gaps in DHTs is the unclear liability landscape, particularly in cases where harm results from data misuse or privacy and confidentiality breaches (Rowland et al. 2022; Seh et al. 2020). Whereas EAPs usually operate within defined liability and accountability structures (which, we grant, can vary greatly between countries), DHTs often exist in a grey area where responsibility is fragmented across multiple stakeholders (Landers et al. 2024). If a DHT results in harm through misdiagnosis, app malfunction, or a privacy breach, determining responsibility is much more difficult due to the diffuse or even absent allocation of responsibility among stakeholders, making it difficult to pinpoint fault when harm occurs (Petersen et al. 2015; Rossmaier et al. 2023)—possibly resulting in a responsibility gap (for in-depth analysis, see Ferlito et al. 2024).

In practice, this ‘gap’ is visible in both Uganda and Malawi. In Uganda, legal reviews of DHTs highlight that responsibility is often shifted onto users through broad disclaimers (‘clickbait’), while enforcement of sector-specific rules remains weak, leaving users without clear avenues for redress when failures and harms occur (Latif 2025). In Malawi, studies of HIV care clients reveal deep concerns about data privacy and confidentiality and lack of transparency over who is accountable when digital systems malfunction or expose sensitive information (Orii et al. 2024). Here, responsibility is diffused across non-governmental organisations (NGOs), donors, vendors, and government actors, making it nearly impossible for users to seek remedy even when harms are serious. These cases show how the absence of enforceable accountability frameworks risks turning DHTs into tools that expand access in the short term but erode trust and entrench systemic vulnerabilities over time.

The EAP model therefore offers one possible source of lessons and inspiration for how to introduce DHTs in LICs. Three important lessons emerge: (a) DHTs should not be presented as definitive solutions, but as a first, imperfect step toward addressing structural and systemic gaps in healthcare access, (b) HCPs and other relevant actors must remain involved so that users are informed about risks and uncertainties and can place trust only in DHTs that genuinely serve patient interests, and (c) regulation and legislation must evolve in sync with the pace of DHT adoption. A broader regulatory framework for ethical AI and data governance must also be enforced, as the rapid growth of DHTs introduces significant ethical concerns (Akinrinola et al. 2024; Morley et al. 2020).

## Recommendations: Toward a Responsible DHT Framework for LICs

If lessons from EAPs are to guide the responsible use of DHTs in LICs, then their implementation, monitoring, and regulation must be rethought. The dilemmas discussed in this paper are not simply about access, nor about choosing between innovation and caution. They stem from deeper structural and systemic conditions that allow DHTs to substitute for long-term investment in healthcare infrastructure. Importantly, LICs are far from homogenous: variations in regulatory capacity, digital infrastructure, and healthcare priorities mean that what works in Malawi may not work in Uganda. A responsible framework must therefore be context-sensitive, avoiding one-size-fits-all solutions that risk flattening diverse realities. What counts as responsible oversight will vary depending on local regulatory capacity, digital readiness, and healthcare priorities of a particular country.

A balanced approach is therefore needed, one that prioritises accountability and prevents regulatory loopholes but does not impose rigid safeguards that block timely access to potentially useful tools. Unlike EAPs, where risks of unregulated use are extreme, responsible DHT oversight in LICs may take a more moderate form: sufficient to protect patients without creating unnecessary bureaucratic hurdles that delay life-improving interventions.

*Here are a few ways this can be done*:

First, privacy, confidentiality, and digital equity must be treated as central design criteria rather than afterthoughts. Without enforceable protections for privacy and confidentiality and proactive measures to address and prevent digital inequalities, DHTs risk eroding the very trust they rely upon. Worse, they may exacerbate disparities by making access contingent on digital literacy, connectivity, or the ability to pay. Any responsible framework must therefore embed privacy, confidentiality, and equity considerations from the outset.

A second step toward responsible implementation requires rejecting the notion that regulatory ‘leniency’ in LICs is a pragmatic necessity. The argument that lower oversight is acceptable in these contexts because DHTs are addressing otherwise unmet needs at times fails to account for the long-term implications of normalising lower standards of healthcare (Hadjiat 2023). Such fragmented efforts not only fall short of sustainable solutions but risk entrenching all sorts of inequalities (e.g. digital inequalities, as only those with mobile connectivity or literacy can benefit).

Yet recent lessons from the COVID-19 pandemic highlight that so-called interim measures rarely remain temporary. Emergency digital rollouts became entrenched not only through inertia but because they aligned with corporate interests and political interests. In this sense, temporary fixes do not just fade away; they risk institutionalising techno-solutionism itself, redirecting scarce resources toward digital stopgaps rather than systemic long-term public investment. This dynamic exacerbates pilotitis and can actively weaken public health infrastructures by normalising fragmented, donor-driven projects as sufficient substitutes for systemic reform.

A different regulatory approach might be needed—one that helps improve healthcare access in LICs but does not treat DHTs as long-term fixes. A tiered regulatory model, inspired by EAPs, would offer a more structured pathway for the introduction of DHTs in LICs. Under such a system, these technologies could be granted conditional approval based on preliminary evidence of efficacy, but this approval would be contingent on ongoing evaluation, post-market surveillance, and clear commitments to refining or replacing suboptimal interventions over time. Instead of viewing regulation as a barrier to innovation, this model would treat it as a continuous process that helps ensure access to healthcare through DHTs does not come at the cost of long-term systemic and structural improvements. As part of this process, regulators could also monitor that DHTs supplement rather than substitute healthcare infrastructure—for instance, by supporting HCPs, building digital literacy, and complementing in-person services rather than excusing underinvestment in facilities.

Third, if DHTs are to function as an ‘interim’ solution, accountability mechanisms must be embedded into their implementation from the outset. At present, the diffusion of responsibility among stakeholders has created a regulatory grey area and somewhat of a ‘responsibility gap’ in which harm often lacks clear pathways for redress. However, responsibility in the context of DHTs cannot be reduced to a legalistic question of liability alone; it must be understood as a broader moral and political obligation (Nissenbaum 1996). Again, Young’s (2011) concept of collective responsibility offers a useful lens. This perspective is especially relevant in LICs, where structural conditions—such as donor-dependence, limited regulation, and private-sector dominance—shape both the harms and the uneven distribution of benefits.

However, as Walker (2006) emphasises, responsibility only carries weight when it is embedded in practices of answerability—processes through which actors can be called to account and required to acknowledge and repair harms; without such practices, responsibility risks dissolving into empty rhetoric rather than producing meaningful change. This means governments must strengthen, not sidestep, regulatory capacity; companies must go beyond rhetoric of ‘empowerment’ and submit to enforceable ethical standards; and donors and NGOs must avoid reinforcing short-term fixes at the expense of long-term system-building.

Fourth, beyond concerns of safety and accountability, the implementation of DHTs in LICs must also be examined through the lens of epistemic trust. In the case of EAPs, users are directly told that the treatments offered are still experimental, which gives them a real chance to weigh the risks before deciding (Raus 2016). By contrast, DHTs are often introduced in LICs under a techno-solutionist narrative that emphasises empowerment and access, without drawing attention to their limitations and potential risks. This narrative risks undermining transparency, hindering informed consent, and risking the erosion of trust in healthcare more broadly, particularly when digital interventions fail to meet expectations or fail to address deeper systemic and structural barriers (Campbell-Verduyn 2021; Sharon 2016).

Another key concern is whether the rapid increase of DHTs will make LICs even more dependent on foreign tech companies and aid, which could make existing global health inequalities much worse. For instance, Birhane (2020) describes this as ‘algorithmic colonisation’: exporting digital health interventions built for HIC priorities into LICs with little adaptation. In Uganda, for example, researchers adapting a mobile phone–based heart failure self-care programme found that despite its promise, numerous rounds of co-design and community engagement were essential to make the tool functional and culturally appropriate for rural users—showing how global empowerment narratives can hide real forms of exclusion (Wali et al. 2023).

Still, rejecting DHTs outright would also be unjust, since it would deprive people in LICs of immediate benefits such as improved health monitoring or teleconsultations where no HCP is available in person. A strict approach, which seeks to heavily regulate all DHTs until ‘perfect’ safeguards are in place, risks becoming another form of injustice, one that withholds access to healthcare from those who could benefit in the present. The challenge, then, is not merely to regulate DHTs, but to do so in a way that maintains a clear commitment to long-term structural reform. It is possible to recognise the immediate value of DHTs in expanding access to care while also resisting the economic and political narratives that seek to present them as an adequate substitute for investment in public health infrastructure.

Ultimately, if DHTs are to be implemented responsibly, then they must be accompanied by clear regulatory pathways, enforceable accountability measures, and commitments to long-term healthcare investment that prevent them from becoming a permanent second-best solution for those who need healthcare the most. If the EAP model offers any lessons for the governance of DHTs, it is that temporary measures can be justified, but only when they operate within frameworks that prioritise patient safety, maintain regulatory integrity, and avoid normalising lower standards of care. The challenge is not merely to use DHTs, but to ensure that their use does not become an excuse for sustaining the very disparities they claim to address.

## Conclusion

The implementation of DHTs in LICs is not a matter of ‘adopt now’ and ‘regulate later’. Access and protection must be pursued together. While DHTs can provide much-needed healthcare access in LICs, rolling them out without safeguards risks normalising lower standards of care and reinforcing systemic and structural inequities. This risk is heightened by digital inequalities, which excludes or disadvantages those without connectivity, literacy, or data affordability, and by unresolved questions of data privacy, confidentiality, and accountability in contexts where protections are weak. Experiences from Uganda and Malawi show that access to DHTs can grow rapidly. However, when their benefits and drawbacks are not carefully assessed, when data protection is not enforced, and when promising tools are not linked to broader improvements in public healthcare—while weaker ones are replaced by structural solutions—projects often fail and public trust erodes.

Lessons from the EAP model suggest that time-limited access can be justified when paired with structured oversight that protects users now and strengthens systems over time. To avoid ‘temporary’ fixes becoming the standard of care, DHT deployment should include a tiered regulatory model, enforceable accountability measures, and long-term commitments to infrastructure development. Crucially, COVID-19 showed how interim solutions can quickly become entrenched through market forces and political expediency, translating into substitutes for public investment rather than complements to it. The central task is therefore to integrate DHTs responsibly: protecting people now, building public capacity for tomorrow, and ensuring digital tools reduce—rather than reproduce—existing disparities in global health.

## Footnotes

1 By ‘LICs’ we refer to the World Bank’s (2025) classification of countries with a gross national income per capita of $1,135 or less.

2 For the purposes of this paper, we use ‘DHTs’ to refer to technologies that patients and citizens engage with, rather than artificial intelligence applications or other digital tools primarily used by HCPs.
